# Activation of Immune System May Cause Pathophysiological Changes in the Myocardium of SARS-CoV-2 Infected Monkey Model

**DOI:** 10.3390/cells11040611

**Published:** 2022-02-10

**Authors:** Maryam Yahya Rabbani, Jay Rappaport, Manish Kumar Gupta

**Affiliations:** 1 Division of Metabolic and Cardiovascular Sciences, Burnett School of Biomedical Sciences, College of Medicine, University of Central Florida, Orlando, FL 32827, USA; maryam.rabbani@ucf.edu; 2 Division of Pathology, Tulane National Primate Research Center, Covington, LA 70433, USA; jrappaport@tulane.edu

**Keywords:** SARS-CoV-2, COVID-19, heart failure, acute respiratory distress syndrome, myocarditis

## Abstract

Severe acute respiratory syndrome coronavirus 2 (SARS-CoV-2) infection is an extremely contagious disease whereby the virus damages the host’s respiratory tract via entering through the ACE2 receptor. Cardiovascular disorder is being recognized in the majority of COVID-19 patients; yet, the relationship between SARS-CoV-2 and heart failure has not been established. In the present study, SARS-CoV-2 infection was induced in the monkey model. Thereafter, heart tissue samples were collected, and pathological changes were analyzed in the left ventricular tissue by hematoxylin and eosin, trichrome, and immunohistochemical staining specific to T lymphocytes and macrophages. The findings revealed that SARS-CoV-2 infection induces several pathological changes in the heart, which cause cardiomyocyte disarray, mononuclear infiltrates of inflammatory cells, and hypertrophy. Furthermore, collagen-specific staining showed the development of cardiac fibrosis in the interstitial and perivascular regions in the hearts of infected primates. Moreover, the myocardial tissue samples displayed multiple foci of inflammatory cells positive for T lymphocytes and macrophages within the myocardium. These findings suggest the progression of the disease, which can lead to the development of severe complications, including heart failure. Additionally, SARS-CoV-2 antigen staining detected the presence of virus particles in the myocardium. Thus, we found that SARS-CoV-2 infection is characterized by an exaggerated inflammatory immune response in the heart, which possibly contributes to myocardial remodeling and subsequent fibrosis.

## 1. Introduction

In the past 24 months, the death toll of a viral pandemic caused by severe acute respiratory syndrome coronavirus 2 (SARS-CoV-2) has surpassed five million worldwide [1]. This coronavirus possesses a positive-sense single-stranded RNA genome and is associated with four major structural proteins: spike, membrane, envelope, and nucleocapsid protein [2]. SARS-CoV-2 infection can cause severe respiratory illness, produce cytokine storm, and even alter gastro-intestinal performance [3]. The current consensus is that people with comorbid conditions, including obesity, hypertension, cardiovascular, and cerebrovascular disorders are predisposed to coronavirus disease 2019 (COVID-19) infection [4]. Interestingly, in addition to respiratory infection, SARS-CoV-2 may perturb cardiovascular functioning, induce myocarditis and hypertrophy, and produce severe complications including arrhythmia, heart failure, acute myocardial infarction, and thromboembolism [5,6,7]. Accumulating evidence suggests that patients suffering from COVID-19 infection have increased levels of plasma troponin I, creatine kinase isoenzyme-MB, and lactate dehydrogenase (LDH) (markers of myocardial injury), which indicate severe cardiac injury secondary to COVID-19 infection [8,9]. Apart from this, echocardiography and magnetic resonance imaging have further elucidated that COVID-19 infection can result in thickening of the ventricular wall, reduced ejection fraction, and myocarditis [10,11].

The ACE2 receptor plays a key role in the attachment, entry, and replication of the virus in the host cell [12]. This receptor is variably expressed in cardiomyocytes, pericytes, fibroblasts, and endothelial cells [13]. Current reports imply that the virus primarily infects pericytes and, later, may cause endothelial dysfunction and disturb cardiovascular homeostasis [13]. It was reported that greater than 7% of COVID patients experience myocardial injury from the infection, which increases to 22% in patients requiring intensive care unit (ICU)-level care [14]. Two distinct patterns of myocardial injury have been proposed, including (1) cytokine storm or secondary hemophagocytic lymphohistiocytosis at 4 days post-symptom onset because of increased troponin, d-dimer, ferritin, interleukin (IL)-6, and LDH; (2) viral myocarditis or stress cardiomyopathy [14]. Despite ongoing clinical research on COVID-19 infection, limited studies are available regarding SARS-CoV-2 infection-dependent pathological changes in the heart [15]. Therefore, there is an urgent need to recapitulate the clinical manifestations of disease using nonhuman primate (NHP) models [16,17]. The present study was envisaged to demonstrate pathological changes in the heart tissue using the monkey model of COVID-19 infection.

## 2. Materials and Methods

### 2.1. Animal Models of Infection and Collection of Biological Tissue Sample

Animal experimental procedures were described before [18,19]. In brief, four adult African green monkeys (AGMs) and four rhesus macaques (RMs) were infected with SARS-CoV-2 (day 0); 2019-nCoV/USA-WA1/2020409; accession number MN985325.1 (Supplementary Table S1). Viral infection was administered through the aerosol route (inhaled dose of 2 × 10^3^ and 2.5 × 10^3^ median tissue culture infectious dose (TCID50)) or multiple routes (cumulative dose of 3 × 10^6^ plaque-forming unit (PFU) virus administered through oral, nasal, intratracheal, and conjunctival routes). Infected animals were euthanized after the development of acute respiratory distress syndrome (ARDS) or when a humane endpoint was reached at 28 days post-inoculation. After necropsy, intact hearts were removed, and an incision was made in the avascular portion of the right and left ventricular free wall. The incisions were extended up through the atria and oracles and the aorta and pulmonary arteries on the left and right sides, respectively. The residual blood was removed, and the chambers were briefly rinsed and dried. Tissue samples were collected from the left ventricular free wall with papillary muscle. Samples were fixed for three to five days in zinc-formalin fixative and processed for paraffin tissue embedding. Formalin-fixed paraffin-embedded (FFPE) tissue sections of 5 µM were prepared for further analysis. For comparison, we used 8 heart samples collected from non-infected monkeys (Supplementary Table S1).

### 2.2. Histological Staining

For histological analysis, cardiac tissue sections were stained with hematoxylin and eosin (H&E) (Sigma, St. Louis, MO, USA). In addition, collagen deposition was detected using a Masson’s trichrome staining kit (Sigma). In brief, cardiac tissue sections were deparaffinized in xylene, rehydrated in graded alcohol to deionized water, and fixed in Bouin’s fixative for one hour at 60 °C. Staining was performed according to the manufacturer’s recommendation, where Weigert’s hematoxylin (nuclei), biebrich scarlet-acid fuchsin (cytoplasm/muscle), and aniline blue (collagen) were used. Slides were mounted with coverslips using Permount mounting medium (Fisher Scientific, Waltman, MA, USA) and scanned using a BZ-X800 Keyence microscope (Keyence, Osaka, Japan).

### 2.3. Immunohistochemistry

Immunohistochemistry (IHC) was performed to characterize myocardial infiltration of inflammatory cells using an ABC immunoperoxidase staining kit (Vector Laboratories, Burlingame, CA, USA). Tissue sections were deparaffinized in xylene, rehydrated in graded alcohol to deionized water, and microwaved for 20 min in 1× citrate buffer for antigen retrieval (Vector Laboratories). Endogenous peroxidase activity was blocked by incubating sections in 3% hydrogen peroxide for 10 min. Nonspecific antibody staining was blocked with 2.5% normal horse serum (Vector Laboratories) for 1 h at room temperature (RT). Slides were incubated with the primary antibodies against macrophages (CD68, clone KP-1 at 1:400 dilution, Dako, Santa Barbara, CA, USA), and T lymphocytes (CD3, polyclonal at 1:600 dilution, Dako) for 1 h at RT. Slides were washed with 1× TBST 3 times for 5 min each. After washing, tissue sections were sequentially incubated with biotinylated anti-rabbit/anti-mouse secondary antibody and avidin/biotin reagent for 30 min each. The sections were developed using 3,3,0-diaminobenzidine (Dako), counterstained with hematoxylin, dehydrated in ascending alcohols to xylene, and coverslipped for bright-field microscopy. For negative control staining, slides were incubated with the IgG-matched primary antibody. 

For SARS-CoV-2 staining, slides were incubated with rabbit anti-SARS-CoV-2 nucleocapsid protein antibody (Novus biologicals, Littleton, CO, USA) at 1:500 dilution for 1 h at RT. To check for nonspecific binding, control tissue sections were stained with the respective antibody. For the negative control, IgG-matched antibody was used instead of primary antibody. Stained tissue sections were scanned using a Keyence microscope (Keyence).

### 2.4. Quantitative Image Assessments

To estimate immune cell infiltrate and the extent of cardiac fibrosis, photomicrographs from three fields stained with H&E and twenty fields stained with Masson trichrome were randomly chosen with the 20× objective of a Keyence microscope, respectively. Images containing large blood vessels were excluded from the study. Inflammation was evaluated by quantifying the cellular nuclei of infected tissue and control tissue sections using ImageJ software, version 1.52b (National Institutes of Health (NIH), Bethesda, MD, USA). Quantification was performed manually to ensure that all blue nuclei were counted once and are reported as the mean number of cells per millimeter square. Fibrosis was quantified as the total area ratio of blue staining/tissue region using a BZ-X800 analyzer software, version 1.1.1.8, and is reported as a mean percentage. For T lymphocyte and macrophage quantification, photomicrographs of 10 regions with the highest CD3+ and CD68+ cells were taken with a 20× objective. Quantitative image analysis was performed by manually counting immunoreactive cells using ImageJ software (NIH). The frequency of positive cells is reported as the mean number of cells per millimeter square.

### 2.5. Statistical Analysis

All statistical analyses were performed using Stata/MP software, version 15 (Stata Statistical Software: Release 15, College Station, TX, USA) using an unpaired Student’s *t*-test with Welch’s correction. All data are expressed as mean ± standard deviation (SD). A *p*-value of less than 0.05 was considered a statistically significant difference.

## 3. Results

### 3.1. SARS-CoV-2 Infection Causes Cardiomyocytes Disarray, Inflammation, and Fibrosis in the Heart

**Figure 1:**
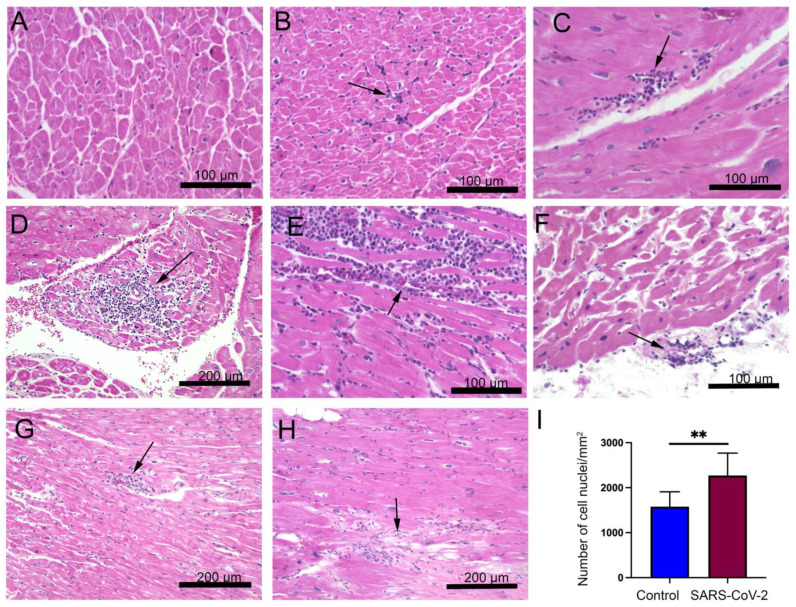
Myocardial lesions are present in the left ventricular cardiac tissue of severe acute respiratory syndrome coronavirus 2 (SARS-CoV-2) infected monkeys. Four adult-aged African green monkeys (AGMs) and four rhesus macaques (RMs) were inoculated with the SARS-CoV-2 virus (day 0) and observed for up to 28 days at the time of necropsy. Healthy monkey hearts (*n* = 8) were used for control. Representative microscopy images show hematoxylin and eosin staining in (**A**) control and (**B**–**H**) SARS-CoV-2 infected hearts (the blue color represents nuclei). (**B**,**C**,**E**) Varying degree of focal mononuclear infiltrates of inflammatory cells with and without necrosis were localized in the interstitium (arrows). (**F**) Focal lymphocytic cells were detected in the pericardium (arrow). (**D**,**G**) Varying degrees of perivascular inflammation manifested in infected monkeys (arrows). (**H**) Myocardial inflammatory cells were associated with areas of tissue degeneration (arrow). (**I**) The graph shows quantification of cellular nuclei in control and SARS-CoV-2 infected hearts (**, *p*-value < 0.01). Scale bars: 200 µm (**D**,**G**,**H**); 100 µm (**A–C**,**E**,**F**).

In our previous studies, we found that SARS-CoV-2 infection generates ARDS in the NHP model of SARS-CoV-2 infection [18,19]. Infected monkeys show minor to early life-threatening symptoms with upregulation of inflammatory cytokines such as IL-6, IL-8, and tumor necrosis factor-alpha [19]. In this study, we examined the pathological changes in the heart tissue of post-mortem animals. A total of eight SARS-CoV-2 infected samples, confirmed with detectable viral load by RT-PCR on nasal and pharyngeal swabs, were included in the study (Supplementary Table S1). AGM 1 and AGM 2 were euthanized subsequent to developing ARDS at 8 and 22 days post-inoculation (DPI), respectively. The remainder of the primates were euthanized at the end of the study, approximately 4 weeks post-inoculation (Supplementary Table S1). An examination of H&E staining revealed that compared to the healthy control, the heart tissue of SARS-CoV-2 infected animals displayed cardiomyocyte disarray, mononuclear infiltrates of inflammatory cells, and necrosis (Figure 1A–I). Diffused and focal aggregates of inflammatory cells were observed in the myocardium of 6/8 (75%) of samples (Figure 1B,C), with extensive focal infiltrates evident in RM3 and RM4 with and without necrosis (Figure 1D,E). Inflammatory cell aggregate in the epicardium of AGM 2 was also appreciated (Figure 1F). Additionally, increased perivascular inflammation (Figure 1D,G) was evident in infected tissue. Aggregates of mononuclear inflammatory cells were found adjacent to interstitial adipocytes, as well as in areas of tissue remodeling (Figure 1H). In contrast, control primates did not display significant inflammatory infiltrates or any degenerative changes (Figure 1A). Quantification of cellular nuclei in SARS-CoV-2 infected and control hearts showed increased cellularity in infected samples, thereby demonstrating an increase in non-cardiomyocyte cells, i.e., inflammatory cells (Figure 1I).

### 3.2. Increased Left Ventricular Fibrosis Detected in SARS-CoV-2 Infected Monkeys

**Figure 2:**
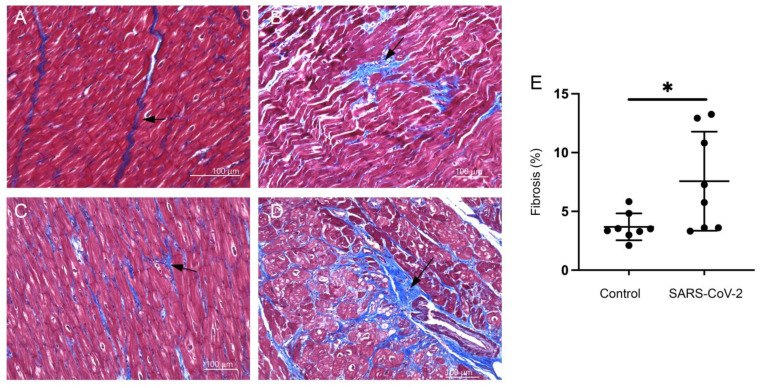
SARS-CoV-2 infected monkeys display increased areas of interstitial and perivascular fibrosis. Masson’s trichrome stain for fibrosis (blue) was done on (**A**) control and (**B**–**D**) SARS-CoV-2 infected hearts. Images show cardiomyocyte disarray and infiltration of fibrotic cells in the left ventricular tissue (**B**), interstitial fibrosis (**C**), and perivascular fibrosis (**D**). (**E**) The graph shows quantitation of fibrotic areas in control and SARS-CoV-2 infected hearts (*, *p*-value < 0.05). Horizontal bars represent mean ± standard deviation (SD). Arrows indicate areas of fibrosis in all figures. Scale bar: 100 µm (**A–D).**

Myocardial fibrotic remodeling was evaluated using Masson’s trichrome stain, revealing interstitial and perivascular fibrosis in infected cardiac tissue (Figure 2A–D). Regions of focal replacement fibrosis were also seen in 3/4 rhesus macaques and 2/4 African green monkeys. Image-analysis-based quantification showed an increased mean percent area of fibrosis in SARS-CoV-2 infected primates compared to the control group (Figure 2E). The majority of infected primates (5/8) had a greater than 5% mean area of interstitial fibrosis in the myocardium.

### 3.3. SARS-CoV-2 Infection Causes Severe Accumulation of Activated T Lymphocytes and Macrophages

**Figure 3:**
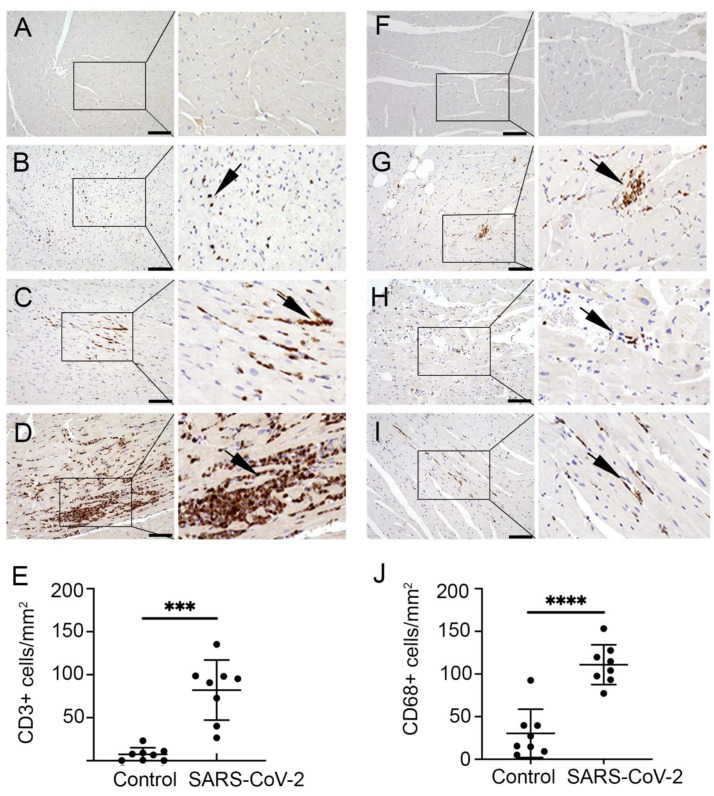
SARS-CoV-2 infected monkeys exhibit increased myocardial T lymphocyte and macrophage infiltration. Representative microscopic images show immunohistochemical (IHC) staining with CD3 antibody on cardiac tissue of (**A**) CRTL 1, (**B**) AGM2, (**C**) AGM4, and (**D**) AGM1. CD3+ cells (brown) were marked diffusely throughout the myocardium (**B**), as well as in varying degrees of aggregates (**C**,**D**). (**E**) Graph shows quantification of CD3 cells in control (mean 7.34 ± 7.72 cells/mm^2^) and infected group (mean 82.16 ± 34.90), ***, *p*-value < 0.001. Images show IHC with CD68 antibody on (**F**) CTRL 1, (**G**) RM1, (**H**) RM2, and (**I**) AGM4. Unlike the control group, macrophages (brown) were observed in small aggregates (**G**), as well as scattered diffusely in the infected group (**H**,**I**). (**J**) Graph shows quantification of CD68+ cells in the control heart (mean 30.44 ± 28.27 cells/mm^2^) and infected group (mean 110.91 ± 23.40), ****, *p*-value < 0.0001. Data are presented as mean ± SD. Arrows indicate CD3 and CD68 positive cells in cardiac tissue. Scale bar: 100 μm.

H&E staining revealed an increased accumulation of immune cells in the infected heart tissue. Hence, the presence of T cells was evaluated by staining with CD3 antibody. The frequencies of CD3+ cell expression and distribution are shown in Figure 3A–J. In the current study, multiple small aggregates of 1–4 foci were noted in all AGMs and three out of four of the infected rhesus macaque models (Figure 3B,C). Focal lymphocytic infiltrates were observed in infected animals (Figure 3D). In addition to the myocardium, T lymphocytes were detected more frequently in the sub-epicardium, epicardium, and fibrotic regions compared to the endocardium. Nevertheless, small aggregates of CD3+ cells were also present in blood vessels and perivascular regions of the endocardium. Mild pericarditis of predominantly CD3+ cells was seen in aerosol (*n* = 1) and multi-route (*n* = 1) infected animals. Quantification data showed that CD3+ cells were significantly increased in the SARS-CoV-2 infected monkeys compared to in the control samples (Figure 3E). The average number of CD3+ cells per 1 mm^2^ was 82.2 (range 26.5–135.3) among infected samples in comparison to the control samples, which had 7.3 cells per 1 mm^2^ (range 0.3–23.4).

Furthermore, interstitial inflammation with the presence of macrophages was characterized using CD68 antibody. Microscopy images showed that CD68+ cells were present diffusely throughout the myocardium as well as in association with fibrotic regions of the infected cardiac tissue (Figure 3F–I). Additionally, aggregates of 1–4 foci were detected in RM1, RM4, AGM1, and AGM4. Unlike T lymphocytes, macrophages were predominant in the subendocardial and myocardial regions and limited in the subepicardium. Increased macrophage accumulation was also observed in blood vessels and perivascular regions. Quantification data showed that CD68+ cells were significantly increased interstitially in SARS-CoV-2 infected primates compared to control samples (Figure 3J). The average number of CD68+ cells per 1 mm^2^ was 110.9 (range 77.3–153.3) in infected tissue compared to 30.4 (range 5.0–92.5) in control samples. A comparison of African green monkeys versus rhesus macaques did not reveal any differences between CD3+ and CD68+ cell infiltrate densities, or in adverse tissue remodeling through fibrosis.

### 3.4. Immunostaining with SARS-CoV-2 Specific Antibody Shows the Presence of Viral Protein in the Heart

**Figure 4:**
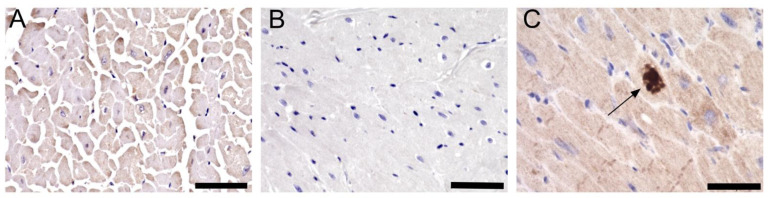
SARS-CoV-2 is localized in the myocardium of infected monkeys. Representative images shows IHC staining done on (**A**) control monkey with the SARS-CoV-2 N protein antibody, (**B**) SARS-CoV-2 infected monkey with IgG control antibody, and (**C**) SARS-CoV-2 infected monkey with the N protein antibody. Arrow shows positive staining (dark brown). Scale bar: 50 µm.

Recent publications showed that SARS-CoV-2 can be detected in the myocardium of infected patients [20,21]. Therefore, the presence of viral infection in the myocardium of SARS-CoV-2 infected primates was investigated using SARS-CoV-2 nucleoprotein antibody. Bright-field microscopy showed the presence of viral protein in the left ventricular myocardium of all eight samples (Figure 4A–C).

## 4. Discussion

COVID-19, caused by SARS-CoV-2, is a respiratory disease that is known to trigger ARDS in patients with severe cases [22]. Despite ongoing research, the characterization of extra-pulmonary manifestation in organs such as the heart through histopathological means has been limited to endomyocardial biopsies and autopsy sections of deceased patients [20,21,23,24,25,26,27,28]. Nonhuman primates (NHPs) are physiologically similar to humans and serve as a valuable model to study cardiovascular disease [29]. To the best of our knowledge, COVID-19-associated myocardial injury in the monkey model has not been extensively explored. The purpose of this study was to evaluate myocardial changes in the left ventricle of monkeys infected through aerosol and multi-route pathways and to characterize the distribution of lymphocytes and macrophages in heart tissue.

In the present study, evidence of pathological changes in the left ventricular myocardium of COVID-19-infected primates is reported. Left ventricular myocardial tissue from these animals contained focal and diffuse mononuclear infiltrates of inflammatory cells with and without cardiomyocytolysis. Additional instances of myocyte hypertrophy and myocardial fibrosis were also evident. Cardiac involvement in COVID-19 patients has been noted in severe infections and implicated as a marker for a worse prognosis [30]. Among the infected animals, aerosol-mediated infected animal AGM1 and multi-route-infected AGM2 developed ARDS and manifested a severe model of disease progression, while the remaining animals exhibited moderate symptoms, suggesting variability in disease progression depending on individual animals [18,19]. Despite this, RM3 and RM4 displayed multi-focal infiltrates of mononuclear inflammatory cells consistent with myocarditis. These findings suggest that myocardial changes can develop in the absence of severe disease.

Cardiac insult is characteristically associated with adverse tissue remodeling and fibrosis secondary to cardiomyocyte death or myocardial degeneration [31]. The current literature reports that myocardial fibrosis may be caused by direct viral involvement and systemic inflammation, leading to long-term disease [32]. In a study of 148 patients, more than half of them displayed late gadolinium enhancement, indicating myocardial fibrosis [33]. Moreover, recent histopathology studies report similar results in the cardiac left ventricle tissue sections of COVID-19 patients [23,32,33]. In our study, myocardial trichrome staining showed a significant increase in interstitial and perivascular fibrosis in both aerosol-infected and multi-route infected monkeys. This is in line with current reports [23,34,35]. At this time, there are no reports of co-staining fibrotic tissue with SARS-CoV-2 antibodies to demonstrate that fibrosis observed is due to the presence of virus particles at those specific areas in the myocardium. Given that most patients in need of hospital care with severe disease usually present with several comorbid conditions, the extent of such findings in relation to COVID-19 remains unclear. In the future, additional testing is needed to link the presence of viral particles with fibrotic regions.

It is established that cells of the mononuclear phagocyte system, such as monocytes and macrophages, help to establish homeostasis in biological processes such as tissue regeneration, innate inflammatory, and adaptive immune responses [36]. In addition to clinical biomarkers data and echocardiographic studies, published reports of COVID-19 cases seldom report positive CD3+ lymphocyte and CD68+ macrophage cell numbers or only report as cells positive per field of view [23,26,28]. Limited studies provide quantification of immune cells per millimeter square in COVID-19 infected patients, which would aid in understanding the degree of the interstitial immune response [27]. The present study describes the distributional patterns and quantification of lymphocytes and macrophages in the cardiac tissue of the COVID-19 NHP model. We found increased interstitial CD3+ lymphocyte infiltration and most focal infiltrates of inflammatory cells, including those found in RM3 and RM4, were CD3+. Increased macrophage count was attributed to elevated systemic levels of proinflammatory cytokines [31]. Here, we report increased myocardial macrophage infiltration in 100% of SARS-CoV-2 infected animals.

It was postulated that myocarditis can occur in healthy uninfected hearts of NHP under human care from catecholamine release. This can lead to vasoconstriction, enhanced myocardial contractility, and ischemia followed by reperfusion, resulting in necrosis and secondary inflammatory response [37]. Nevertheless, the higher rate of myocarditis in viral infection may result from a direct toxic effect, chronic immune stimulation leading to persistent inflammation, opportunistic infections, or an autoimmune response [37].

In this study, we report the presence of SARS-CoV-2 in the left ventricular myocardium of all eight samples obtained from COVID-19-infected primates. The number of SARS-CoV-2 positive cells was very low, which were distributed throughout the cardiac tissue. These findings are in agreement with other reports [25,28]. In a study by Lindner et al., SARS-CoV-2 localization was observed in interstitial cells and macrophages through RNA scope in 16 autopsy cases of COVID-19 patients [20]. Whether these are resident macrophages that were directly infected by SARS-CoV-2 in the heart or if they migrated from the lungs into extra-pulmonary tissues is still unclear [38]. Thus far, reports of SARS-CoV-2 infected cells in the heart tissue of COVID-19 cases have been variably presented. Despite multiple studies demonstrating viral RNA presence in myocardial tissue, heterogeneous presentations in infected cardiac tissue are seen [22,23,39]. In a study by Fox et al., using electron microscopy, they found that of the 22 patients who died from COVID-19, SARS-CoV-2 particles were present in the endothelial compartment of the hearts [39]. Another report suggested direct SARS-CoV-2 viral infection in an 11 year old girl with myocarditis and HF in several lineages of the heart, including cardiomyocytes, endothelial cells, mesenchymal cells, and inflammatory cells [27]. Lastly, a case study by Gauchotte et. al., of a 69 year old man who died from refractory shock and multi-organ failure, demonstrated through IHC the presence of SARS-CoV-2 nucleoprotein in cardiomyocytes but not in the lung tissue [22]. This study contributes to the current literature regarding the variable presentation of myocardial injury and describes one such possible means of infection.

## 5. Conclusions

Our main findings were increased infiltration of mononuclear cells positive for CD3 lymphocytes and CD68 macrophages with and without myocyte injury. In addition, the presence of SARS-CoV-2 nucleoprotein in infected myocardium correlates to clinical presentation in COVID-19 patients. Overall, cardiac pathological changes associated with SARS-CoV-2 are heterogeneous in nature. These findings contribute to further studies regarding the cardiotropic potential of SARS-CoV-2 and its histopathological manifestation.

## 6. Limitations

This study has several limitations. First, due to the limited availability of tissue samples, molecular analysis for the virus in the myocardium using RT-PCR was not performed. Second, functional cardiovascular tests such as echocardiograms or cardiac magnetic resonance imaging (MRI) scans were not performed at the time of the study. Third, given that the samples were collected from a study focusing on respiratory changes in NHPs infected with SARS-CoV-2, special testing for cardiac biomarkers was not carried out in routine serum collection. Lastly, exclusion of coinfection with opportunistic viruses of cardiotropic nature was not employed for similar reasons.

## Data Availability

The data presented in this study are available upon request from the corresponding author.

## Supplementary Materials

The following supporting information can be downloaded at: https://www.mdpi.com/article/10.3390/cells11040611/s1, Supplementary Table S1: Animal route of infection, dosage, demographic information, and time of necropsy.
