# Supplementary material for: Activation of Immune System May Cause Pathophysiological Changes in the Myocardium of SARS-CoV-2 Infected Monkey Model
Source: Cells. 2022 Feb 10;11(4):611. doi: 10.3390/cells11040611 (PMC8869860; doi:10.3390/cells11040611)
Supplement: Supplementary file 1 [file cells-11-00611-s001.zip › cells-1537444-supplementary.pdf]

cells

MDPI

Supplementary Material

Table S1. Animal route of infection, dosage, demographic information, and time of necropsy.

No.	Animal	Infection Route	Dose	Age	Sex	Necropsy (DPI)
1	RM1	Multi-route	3.61e+06 PFU	14	M	27
2	RM2	Multi-route	3.61e+06 PFU	13	F	27
3	AGM 2	Multi-route	3.61e+06 PFU	16	F	22 (ARDS)
4	AGM 3	Multi-route	3.61e+06 PFU	16	M	26
5	RM3	Aerosol	1.0e+04 PFU	13	M	28
6	RM4	Aerosol	1.0e+04 PFU	15	M	28
7	AGM 1	Aerosol	1.0e+04 PFU	16	F	8 (ARDS)
8	AGM 4	Aerosol	1.0e+04 PFU	16	M	24
9	CTRL1	-	-	3	F	
10	CTRL2	-	-	3	F	
11	CTRL3	-	-	3	F	
12	CTRL4	-	-	5.39	F	
13	CTRL5	-	-	6	M	
14	CTRL6	-	-	10	F	
15	CTRL7	-	-	10	M	
16	CTRL8	-	-	7	F	
